# A Low-Cost and Unsupervised Image Recognition Methodology for Yield Estimation in a Vineyard

**DOI:** 10.3389/fpls.2019.00559

**Published:** 2019-05-03

**Authors:** Salvatore Filippo Di Gennaro, Piero Toscano, Paolo Cinat, Andrea Berton, Alessandro Matese

**Affiliations:** ^1^Institute of Biometeorology, National Research Council (CNR-IBIMET), Florence, Italy; ^2^Institute of Clinical Physiology, National Research Council (CNR-IFC), Pisa, Italy

**Keywords:** UAV, computer vision, high throughput field-phenotyping, yield estimation, unsupervised detection

## Abstract

Yield prediction is a key factor to optimize vineyard management and achieve the desired grape quality. Classical yield estimation methods, which consist of manual sampling within the field on a limited number of plants before harvest, are time-consuming and frequently insufficient to obtain representative yield data. Non-invasive machine vision methods are therefore being investigated to assess and implement a rapid grape yield estimate tool. This study aimed at an automated estimation of yield in terms of cluster number and size from high resolution RGB images (20 MP) taken with a low-cost UAV platform in representative zones of the vigor variability within an experimental vineyard. The flight campaigns were conducted in different light conditions and canopy cover levels for 2017 and 2018 crop seasons. An unsupervised recognition algorithm was applied to derive cluster number and size, which was used for estimating yield per vine. The results related to the number of clusters detected in different conditions, and the weight estimation for each vigor zone are presented. The segmentation results in cluster detection showed a performance of over 85% in partially leaf removal and full ripe condition, and allowed grapevine yield to be estimated with more than 84% of accuracy several weeks before harvest. The application of innovative technologies in field-phenotyping such as UAV, high-resolution cameras and visual computing algorithms enabled a new methodology to assess yield, which can save time and provide an accurate estimate compared to the manual method.

## Introduction

Grapes are one of the most widely grown fruit crops in the world: vineyards cover a total area of 7.5 million hectares and produce a total yield of 75.8 million metric tons, of which 36% are fresh grapes, 8% raisins and 48% wine grapes (International Organisation of Vine and Wine [OIV], 2017).

Monitoring and grading in-field grape ripeness and health status is extremely important for valuable production, for both the table grape and premium wine markets (Bindon et al., 2014; Ivorra et al., 2015; Portales and Ribes-Gomez, 2015; Pothen and Nuske, 2016). As for other crops, the yield monitoring in terms of cluster number and size is key information in viticulture (Fanizza et al., 2005; Cabezas et al., 2006; Costantini et al., 2008). Traditionally, the yield prediction is conducted manually and routinely evaluated by visual or destructive methods, like those proposed by the International Organisation of Vine and Wine [OIV] (2007), which may involve problems such as low efficiency in terms of time and sampling representativeness (Pothen and Nuske, 2016). Moreover, yield evaluation with visual inspection by means of cluster counting and size estimation is subjective resulting in error variations between the results of different people (Roscher et al., 2014).

Field conditions, frequently on a slope and plowed soil, cause measurement difficulties, especially in the summer due to extremely high temperature. In that respect, advances in technology are key to the future of agriculture. Computer vision is a powerful tool for measuring dimensions and size distribution of shaped particles. Specific user-coded computer vision applications of particle size may require advanced programming using a proprietary programming language environment such as Visual C or MATLAB with specialized image processing toolboxes (Igathinathane et al., 2008). ImageJ is a Java-based, multithreaded, freely available, open source platform, independent and public domain image processing and analysis program developed at the National Institutes of Health (NIH), United States (Schneider et al., 2012). ImageJ has a built-in option for analyzing particles, which produces output parameters such as number of particles, areas, perimeters, and major and minor axes. In our study, the shaped particles are vine clusters. This software provides many image analysis tools, including algorithms based on threshold detection value to generate the binary image used for segmentation. Otsu’s threshold is one of the most widely used threshold techniques for the vegetation segmentation process (Ling and Ruzhitsky, 1996; Shrestha et al., 2004). Gebhardt et al. (2006) converted the RGB images into grayscale generating local homogeneity images to detect a homogeneity threshold.

The main limitation of threshold techniques is stability of the binarization accuracy of the system, as any mis-segmentation is generally caused by an error in the detected threshold. So, if the detected threshold is not appropriately estimated, the generated segmentation process will be strongly affected. Another issue is related to the effect of light conditions on the vegetation segmentation results obtained, particularly in sunny and overcast conditions. The use of different color spaces instead of RGB can overcome the unwanted light effects. A widely used color model is the LAB color model, whose coordinates represent the lightness of the color (L^∗^), its position between magenta and green (a^∗^) and between yellow and blue (b^∗^). The LABFVC algorithm proposed by Liu et al. (2012) is an automatic Fractional Vegetation Cover (FVC) extracting algorithm for digital images and is based on the premise that the representations of vegetation and soil in the LAB color spaces approximately follow Gaussian distributions. Song et al. (2015) proposed a modified methodology based on LABFVC algorithm as automatic shadow-resistant algorithm in the LAB color space (SHAR–LABFVC). Yang et al. (2015) suggested an HSV (hue, saturation, value) color space method for greenness identification of maize seedling images acquired outdoors.

Computer vision has also been widely applied for size and weight evaluation because it is non-destructive and highly efficient (Costa et al., 2011; Cubero et al., 2011; Hao et al., 2016). In agriculture and the food industries, due to recent advances in computing and robotics, most computer vision applications are related to the measurement of external properties, such as color, size, shape and defects of fruits, vegetables, fish and eggs (Zhang et al., 2014; Soltani et al., 2015; Moallem et al., 2017; Su et al., 2017).

The literature reports several attempts, with the use of computer vision, to characterize morphological attributes of grapes (Wycislo et al., 2008; Miao et al., 2012), to evaluate different cluster and berry traits (Kicherer et al., 2013; Cubero et al., 2014; Diago et al., 2014; Roscher et al., 2014; Tello and Ibanez, 2014; Aquino et al., 2017) and to assess the number of grapevine flowers per inflorescence (Diago et al., 2014; Aquino et al., 2015).

Recently, research has focused on estimating grape yield components using stereo vision (Ivorra et al., 2015) and comparing 2D imaging technology with direct 3D laser scanning system (Tello et al., 2016). The latter successfully achieved a geometric reconstruction of the morphological volume of the cluster from 2D features, which also proved to work better than the direct 3D laser scanning system. All these studies were conducted in the lab on grape samples collected manually, while image processing has been used successfully in the field with prototype rovers or tractors to assess key canopy features, such as yield (Dunn and Martin, 2004; Nuske et al., 2011; Diago et al., 2012; Nuske et al., 2014; Aquino et al., 2018) and leaf area (Tardaguila et al., 2012). Moreover, recent papers (Herzog et al., 2014; Roscher et al., 2014; Kicherer et al., 2015) have reported some initial results on the application of image analysis for high-throughput phenotyping in vineyards. Those solutions demonstrated high performance and weak points such as the long monitoring time due to slow forward speed and problems related to soil trafficability. Furthermore, those solutions enhanced image quality using artificial light in the night time condition, with all the related risks for operators in a sloping vineyard or in narrow vineyard rows.

The above papers proved that this new framework, based on computing-robotics-machine vision, can be applied successfully for the evaluation of cluster attributes and components. In addition, precision viticulture is experiencing substantial growth due to the availability of improved and cost-effective instruments such as UAVs (Unmanned Aerial Vehicles; Matese et al., 2015). It has been demonstrated that rapid technological advances in unmanned aerial systems foster the use of these systems for a plethora of applications (Gago et al., 2015; Pôças et al., 2015; Bellvert et al., 2016; Di Gennaro et al., 2016; Poblete-Echeverría et al., 2017; Romboli et al., 2017; Santesteban et al., 2017; Matese and Di Gennaro, 2018), opening also new perspectives to traditional remote sensing (Sun and Du, 2018).

The aim of this study was to develop a fast and automated methodology to provide an early yield evaluation (pre-harvest) in support of vineyard management. The UAV approach described allows a fast monitoring taking into account a few images acquired in representative points of vineyard variability, which is fundamental to export this application to a large vineyard with moderate times for monitoring and image processing. This method aims to provide useful information, overcoming the limits of ground observations (soil trafficability and forward speed). To our knowledge, this is the first study to apply these technologies in a partially defoliated vineyard with vertical pruning system (VPS), while a first attempt was successfully applied to detect melons in pre-harvest (Zhao et al., 2017). The proposed methodology was evaluated with high resolution RGB images acquired by UAV in a vineyard located in Tuscany (Italy), with a commercial digital camera and without the help of artificial light.

## Materials and Methods

### Case Study and Experimental Design

**FIGURE 1:**
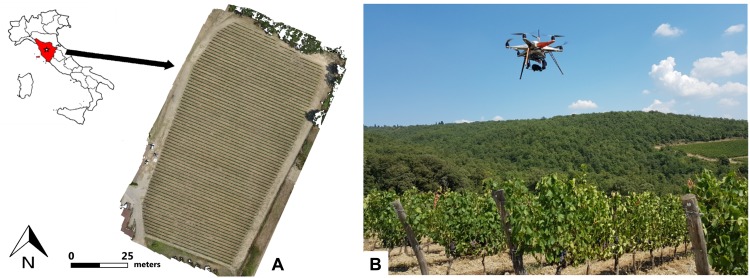
Field location map **(A)** and UAV platform **(B)**.

The research was undertaken during 2017 and 2018 seasons in a 1.4 ha vineyard (43°25′45.30″N, 11°17′17.92″E) planted in 2008 and located in Castellina in Chianti (Chianti domain, central Italy; Figure 1A). Sangiovese cv. (Vitis vinifera) vines were trained to a vertical shoot-positioned trellis and spur-pruned single cordon with four two-bud spurs per vine. Vine spacing was 2.2 m × 0.75 m (inter-row and intra-row), rows were NW-SE oriented, and the vineyard was on a slight southern slope at 355 m above sea level. Pest, soil and canopy management were performed following the farm practices.

A preliminary flight campaign on the vineyard was performed with a UAV platform on June 27th 2017, which is a key period in this winemaking area for estimation of vine vigor variability (Romboli et al., 2017). Remote sensing multispectral images were collected in order to characterize vigor spatial variability and identify representative vigor zones for the grape detection analysis. Within the representative zones of high and low vigor, five vines were selected in each zone. On August 9th those vines were monitored by a UAV equipped with an RGB camera to assess tool performance in cluster detection in two different light conditions combined with two different leaves management: (i) target shaded by leaves and shadow, (ii) target partially free of leaves and directly illuminated by the sun. Those conditions were evaluated performing a flight before (i) and after (ii) a partial defoliation, which was done by removing the leaves in the fruiting zone from just one side of the row, to maintain partial cluster protection and not excessively alter the vegetative-productive balance of plants. This is a widespread agronomic practice in order to favor grape ripening and health, limiting the risk of summer heat stress. Two datasets were acquired, the first in the morning (10:00 AM) in worst condition (W), with cluster covered by leaves and in shadow; and the second in the afternoon (14:00) in best condition (B), with leaves partially removed and cluster directly illuminated by the sun. On the same day, a third UAV survey was conducted with the aim of confirming the distribution variability detected during the first flight in June. The same flight campaigns as 2017 were repeated on June 26th and August 8th 2018. The experimental design was modified according the preliminary results obtained in 2017. So, the acquisition was conducted by choosing the best performing method of the two tested, expanding the number of experimental parcels. In detail, two blocks per vigor area of eight plants each were identified, for a total of 32 sample plants.

### Ground-Truth Measurements

In 2017 season, the characterization of vigor variability within the vineyard was performed taking into account 18 vines in each vigor zone: five sample vines monitored in detail by UAV and 13 chosen randomly around sample vines. In 2018 season, ground truth measurements were taken on 16 sample vines monitored in detail by UAV for each vigor zone. For each vine, vegetative and yield-related data were monitored. As indicator of vine vigor, total shoot fresh mass was determined in the field for each vine in the dormant period, prior to pruning. In both years, the production of each sample vine was characterized in the field in terms of cluster number (ripe and unripe) and yield.

### UAV Platform and Sensors

Flight campaigns were performed using an open-source UAV platform consisting of a modified multi-rotor Mikrokopter (HiSystems GmbH, Moomerland, Germany; Figure 1B).

Autonomous flight is managed by an on-board navigation system, which consists of a GPS module (U-blox LEA-6S) connected to a navigation board (Navy-Ctrl 2.0) and a flight control unit (Mikrokopter Flight Controller ME V2.1) that controls six brushless motors. Two communication systems consist of a duplex transmitter at 2.4 GHz (Graupner) and a WiFi module (Mikrokopter) at 2.4 GHz to control the UAV navigation and monitor flight parameters, while a WiFi module provides video data transmission at 5.8 GHz ensuring real-time image acquisition control by the ground operator. The flight planning was managed through Mikrokopter Tool software, which allows a route of waypoints to be generated as a function of the sensor Field Of View, overlap between images and ground resolution needed. Maximum payload is approximately 1 kg, ensuring 15 min of operating time with one 4S battery @11000 mAh. A universal camera mount equipped with three servomotors allows an accurate image acquisition through compensation of tilt and rolling effects.

The UAV was equipped with multispectral and RGB cameras, to monitor vegetative status and perform image detection, respectively. The multispectral camera ADC-Snap (Tetracam Inc., CA, United States) mounts a global shutter CMOS sensor that acquires 1280 × 1024 pixel images (1.3 MP) in the green (520–600 nm), red (630–690 nm), and near-infrared (760–900 nm) bands. Multispectral image acquisition was performed at 50 m above the ground, yielding a ground resolution of 0.025 m/pixel and 70% of overlap in both directions. The RGB camera is a Sony Cyber-shot DSC-QX100 RGB camera (Sony Corporation, Tokyo, Japan), which mounts a 20.2 megapixel CMOS Exmor R sensor and a Carl Zeiss Vario–Sonnar T lens. The RGB camera acquired high resolution data of sample plants selected within representative zones of the vineyard variability. The flight altitude was set at 10 m above the ground and the sensor was placed at 45° with respect to the vertical at the ground and 90° with respect to the direction of advancement, providing a ground resolution of 0.002 m/pixel on the fruit zone. Flight planning was made by choosing a route of waypoints following the direction of the rows, which allows images of the area of interest on adjacent rows to be acquired.

### Multispectral Data Processing

PixelWrench2^1^ software provides color processing of Tetracam RAW files and then radiometric correction, thanks to a calibration target acquired before each remote-sensing campaign. The images were subsequently processed in a series of steps with Agisoft Photoscan Professional (v.1.4.1),^2^ a commercial computer vision software package, and Quantum GIS,^3^ an open source GIS software, to provide a vigor map (Matese et al., 2015). The filtering procedure of the pure canopy pixels was assessed with a digital elevation model output produced from Agisoft Photoscan software. The basis of the procedure was that vine rows have a greater height from the ground and can easily be discriminated by Otsu’s global thresholding, an algorithm that allows discrimination of two different zones: vine rows and ground (Matese et al., 2016). Assuming the correspondence between NDVI and vigor (Costa-Ferreira et al., 2007; Fiorillo et al., 2012), the first step is derivation of the NDVI computed by the following equation:

NDVI=(Rnir−Rred)/(Rnir+Rred)

where Rnir and Rred are the reflectance in near infrared and red bands (Rouse et al., 1973). Matlab software (v.7.11.0.584, 2010)^4^ was used to interpolate pure canopy pixel values with a moving average window and elaborate a NDVI map.

### Computer Vision and Machine Learning: From RGB to Cluster Yield Workflow

**FIGURE 2:**
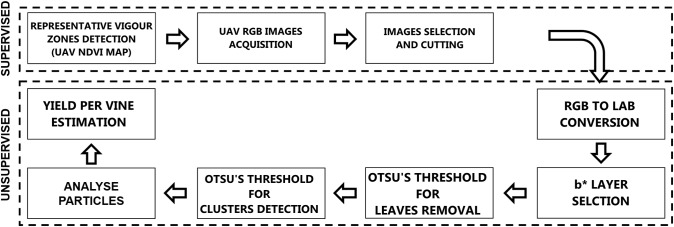
Process flowchart.

Digital images acquired by UAV camera are stored in three dimensions using RGB color space and for color pattern extraction like vegetation, background or other components; color images offer more dimensions for image segmentation. Analyzing the distribution curve for different components permits the classification by determining a threshold in one-dimensional space. Our methodology is completely unsupervised using ImageJ software and a threshold function with Otsu’s method. Image processing essentially involves the creation of binary images of the particles from RGB images before being processed by the application algorithm. Figure 2 shows the methodology flowchart.

#### Selection of RGB Images

**FIGURE 3:**
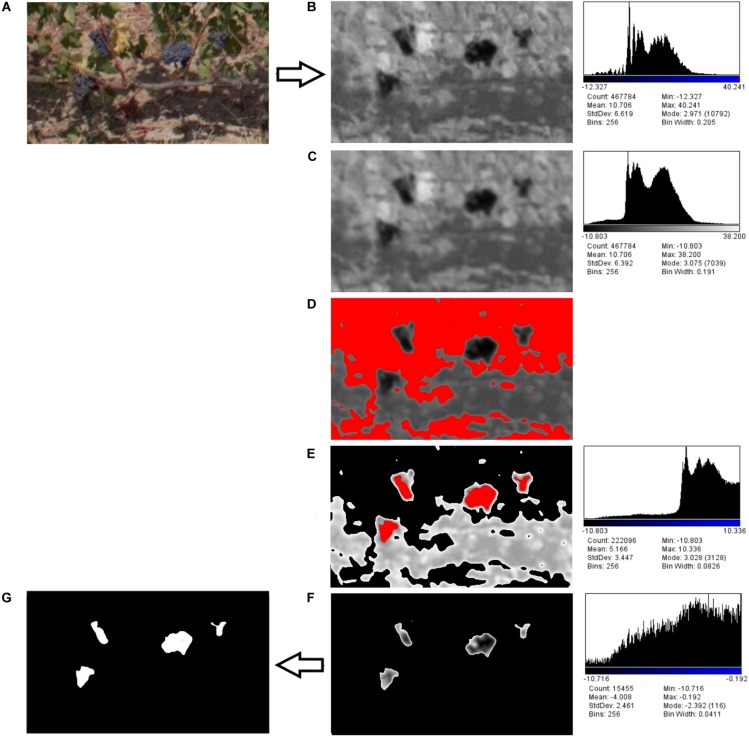
Detailed image processing workflow: **(A)** RGB supervised image selection, **(B)** component b^∗^ from RGB to LAB conversion, **(C)** Gaussian filter, **(D)** first Otsu’s threshold for leaves detection, **(E)** vegetation pixels removal, **(F)** second Otsu’s threshold for clusters detection, **(G)** mask conversion.

The first step of RGB images analysis workflow was the “supervised” selection of images centered on the sample plants in different conditions for each vigor zone (Figure 3A).

#### Lab Stack Conversion

Initially RGB was converted to Lab Stack using ImageJ standard commands. The Lab color space mathematically describes all perceivable colors in the three dimensions: L for lightness, a^∗^ and b^∗^ for the color opponents green–red and blue–yellow. The yellow/blue opponent colors are represented along the b^∗^ axis, with blue at negative b^∗^ values and yellow at positive b^∗^ values. The component b^∗^ was found to be strictly correlated with cluster color (Figure 3B). A Gaussian filter with radius equal to 2 was applied in order to enhance the distribution function(histogram; Figure 3C).

#### Thresholding

The thresholding step was tried with different images and Otsu’s method was chosen to locate a threshold value automatically based on each image’s condition. Otsu’s threshold clustering algorithm searches for the threshold that minimizes the intra-class variance, defined as a weighted sum of variances of the two classes. The algorithm assumes that the image contains two classes of pixels following a bi-modal histogram (foreground and background pixels), it then calculates the optimum threshold separating the two classes so that their combined spread (intra-class variance) is minimal, or equivalent (because the sum of pairwise squared distances is constant), so that their inter-class variance is maximal.

Two threshold routines were applied sequentially to b^∗^ filtered images. The first routine applying the automatic Otsu’s threshold in order to identify the pixels considered as vegetation (Figure 3D). Those pixels were removed from the images (Figure 3E) and a new histogram of the image was obtained. The second routine with a second Otsu’s threshold automatically detected only the clusters (Figure 3F); the cluster values were then converted into a mask (Figure 3G).

#### Analyze Particles

The next step was to set the scale by drawing a line on a known length target, so as to enable the tool in the pixel count along the drawn line on spatially calibrated images. ImageJ “Analyze Particles” routine was then invoked, which generates the number and dimensions of the particles (clusters). In the “Analyze Particles” dialog, particle areas to be considered were set at 200 to infinity pixel units, covering only cluster dimension, circularity was set at 0.25–1.00 and Overlay mask to display the number of shapes.

#### Statistical Analysis

The classification performance of the unsupervised methodology described was evaluated through a sensitivity index or true positive rate (TPR):

TPR=(true cluster automatically classified/total clusters observed)/100.

The TPR identifies the percentage ratio of the true cluster automatically classified and the number of total clusters observed. Instead, the accuracy of the UAV approach was assessed by the value of a Percent accuracy index:

Percent Accuracy=[(measured value-estimated value)/measured value]×100.

The Percent Accuracy is calculated by subtracting the estimated value from the measured one, dividing that number by the measured value and multiplying the quotient by 100.

#### Yield Estimation

Yield estimation was performed by using the clusters surface derived from image analysis and the yield per vine weighted by traditional ground sampling. As a first step, for 2017 season, a linear regression was obtained between clusters surface (cm^2^) and grapes sampled (g) on representative vines in both high and low vigor areas. Following 2017 results, the linear regression parameters were applied on 2018 dataset to calculate yield from remote sensing data, which was finally validated against ground truth measurements.

### Cost-Benefit Analysis

The cost analysis was applied to three ideal vineyard sizes: 5, 10 and 50 ha, according with the last European Farm structure survey (Farm indicators by agricultural area, farm type, standard output, legal form and NUTS 2 regions; Eurostat, 2018). The approach was adopted to account for all the expenses associated to data acquisition and processing, for both the proposed methodology and the traditional survey, grouped into four broad categories, plus the cost for equipment (UAV and RGB camera) purchase:

- Survey timing, i.e., the time needed to make a survey in two zones per hectare. For the traditional ground survey, it was calculated as about 25 min/ha to move within the vineyard, count and measure the average yield (10 vines). For the UAV 3 min/ha was estimated: 1 min for take-off and landing and 2 min for image acquisition.

- Survey costs include the man-hour costs to monitor the vineyard in a traditional way and to perform a UAV data acquisition flight. The cost for a single man-hour was considered at $16/h for a skilled worker who undertakes the traditional survey and at $24/h for a trained UAV pilot.

- Elaboration timing takes into account the digitization of the observed and measured data in the field (2 min/ha), while for the proposed UAV methodology it is the time to select the single images of interest and perform the automatic recognition (10 min/ha).

- Elaboration costs include the man-hour costs to digitalize the data acquired by the skilled worker with a related cost of $16/h for ground data and $20/h for image analysis.

- Cost of UAV+RGB camera, based on a DJI Phantom 3 platform + 4K 12 Megapixel camera purchase with a 3 years depreciation cycle ($620 total cost, $17.2 per month in depreciation costs) and assuming only one survey per year.

All the reported costs are provided by Payscale, a website that provides information about salary, benefits and compensation,^5^ while the costs of UAV+RGB are readily available on the internet (Google Shopping, accessed September 30, 2018).

## Results

### Representative Zones

**FIGURE 4:**
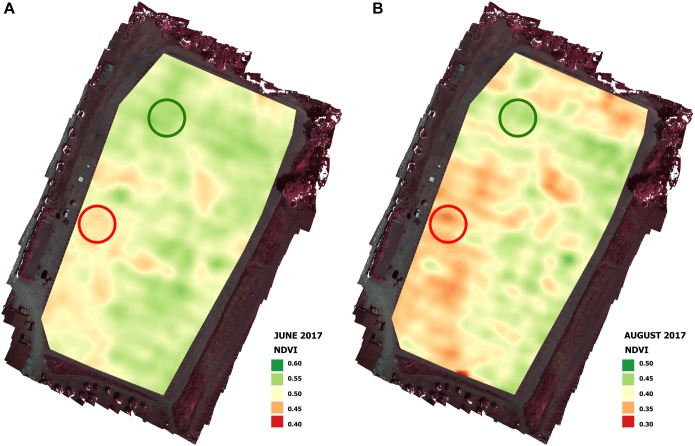
Vigor maps of June **(A)** and August **(B)** flight campaigns in 2017 season with representative high vigor (HV) and low vigor (LV) zones.

Calculation of the NDVI values allowed the identification of two representative zones of the vigor heterogeneity within the vineyard (Figure 4): one representative of the HV zone (high vigor) and the other of the LV zone (low vigor). The vigor within the vineyard detected during the preliminary flight in June 2017 (Figure 4A) was confirmed by the vigor map produced by the flight campaign in August 2017 (Figure 4B).

Remote sensing data related to vigor variability within the vineyard were confirmed by ground measurements for vegetative data acquired through the sampling of 18 vines in each zone identified by UAV survey (Table 1).

**Table 1:** Remote sensing and ground truth vine vegetative assessments extracted in representative high (HV) and low vigor (LV) zones.

	HV	LV	Student’s *t*-test
NDVI June 2017	0.54 ± 0.05	0.49 ± 0.04	^∗∗∗^
NDVI August 2017	0.43 ± 0.08	0.36 ± 0.09	^∗∗∗^
Shoot fresh mass 2017 (kg)	0.39 ± 0.10	0.16 ± 0.04	^∗∗∗^
NDVI June 2018	0.58 ± 0.02	0.54 ± 0.04	^∗∗^
NDVI August 2018	0.60 ± 0.03	0.53 ± 0.05	^∗∗∗^
Shoot fresh mass 2018 (kg)	0.50 ± 0.13	0.31 ± 0.08	^∗∗∗^


*Values are the mean ± SD. Statistical difference was assessed by Student’s *t*-test: ^∗∗∗^*P* < 0.001; ^∗∗^*P* < 0.01.*

### Cluster Characteristics Results

**FIGURE 5:**
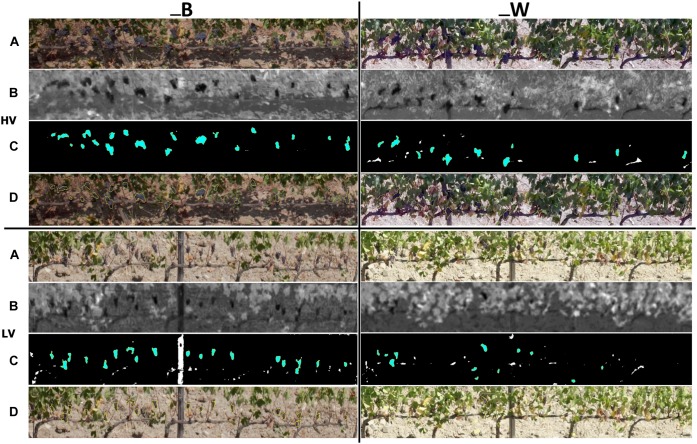
Image analysis output for cluster detection within high (HV) and low (LV) vigor zones in best (_B) and worst (_W) conditions of image acquisition in 2017 season: **(A)** extraction of sampling vines from raw RGB image, **(B)** LAB image processing, **(C)** automatic cluster detection, **(D)** RGB image with cluster overlay mask.

The results of cluster detection in Worst condition (W) – target partially covered by leaves and shaded, Best condition (B) – target partially free of leaves and directly illuminated by the sun are shown in Figure 5, which depicts the workflow of the image processing steps in HV (left) and LV (right) zones performed with ImageJ software. Figure 5 shows the visual results of 2017 season related to cluster characteristics detection from raw RGB images acquired by UAV (Figure 5A), LAB images processing (Figure 5B), automatic cluster detection (Figure 5C) and lastly RGB images with a cluster overlay mask (Figure 5D). In 2017, the one side defoliation left very little number of leaves in low vigor zone as a consequence of the minimum leaf coverage due to extremely dry season.

The results provided by image analysis algorithm performed with the ImageJ software in B and W conditions within the two vigor zones are summarized in Table 2. Regarding the results of the 2018 season, only the best condition was taken into account on a larger sampling number of plants.

**Table 2:** Cluster detection performance methods on sample vines in high (HV) and low vigor (LV) zones in best (_B) and worst (_W) conditions.

	2017				2018	
		
	HV_B	LV_B	HV_W	LV_W	HV_B	LV_B
**Observed**						
Clusters per vine	6.0 ± 2.0	4.8 ± 1.5	6.0 ± 2.0	4.8 ± 1.5	7.1 ± 3.2	5.6 ± 3.2
Green Clusters per vine	0.8 ± 0.8	0.6 ± 0.6	0.8 ± 0.8	0.6 ± 0.6	1.9 ± 1.2	1.8 ± 1.3
**UAV**						
Clusters per vine UAV	4.0 ± 1.0	3.6 ± 1.5	2.2 ± 0.8	1.2 ± 1.3	2.6 ± 1.5	2.9 ± 1.9
Clusters per vine UAV ADJ	5.2 ± 1.8	4.2 ± 1.8	3.0 ± 1.9	1.4 ± 1.7	4.7 ± 2.4	4.1 ± 2.7
TPR (%)	79.6	87.1	43.5	23.6	65.7	81.0
TPR_Ripe (%)	100.0	100.0	54.9	26.4	84.8	97.7


*The mean ± standard deviation values of: Clusters per vine - number of clusters per vine monitored by ground observation; Green Clusters per vine - unripe clusters; Clusters per vine UAV - clusters detected by UAV; Clusters per vine UAV ADJ - number of clusters detected by UAV adjusted taking into account the presence of double clusters. TPR and TPR_Ripe is the true positive rate related to total and ripe clusters.*

For each vigor × condition, Table 2 reports the mean and standard deviation of number of clusters per vine monitored by ground observation (Clusters per vine), presence of green clusters (Green Clusters per vine), clusters detected by UAV (Clusters per vine UAV), number of clusters detected by UAV adjusted taking into account the presence of oversized clusters assessed as double clusters (Clusters per vine UAV ADJ). This was performed applying a threshold on the cluster dimension monitored by UAV, which allowed the presence of two very close clusters to be detected, first identified as one oversized cluster. The UAV methodology performance in different conditions of vigor and image quality was calculated through a TPR index. The UAV methodology applied in HV_B and LV_B identified 100.0% of ripe grapes in 2017 season, while poorer performances were found with HV_W (54.9%) and LV_W (26.4%). The image analysis of 2018 season provided a lower performance in single cluster segmentation on ripe grapes with HV_B (84.8%) and LV_B (97.7%).

### Yield Estimation

Following these results, the data obtained from the best performing method (_B) were used to calculate the production values per plant in each vigor zone in both seasons.

The zonal statistics calculated with ImageJ allowed the clusters to be counted and sized, processing the surface area exposed by the 2D image acquired by UAV. The total values of cluster area per vine extracted on high (168.6 ± 84.0 cm^2^) and low (69.2 ± 35.6 cm^2^) vigor plants on 2017 season were converted by cluster weight through a linear regression with the ground truth measurements.

**FIGURE 6:**
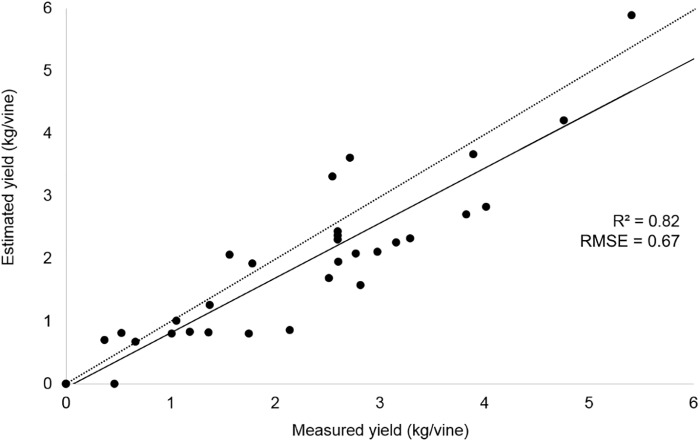
Correlation between yield measurements in the field and yield estimation from UAV image analysis approach related to 2018 season.

The yield per vine was computed from the 2018 season dataset, according to the correlation parameters provided by 2017 preliminary results (*R*^2^ = 0.90) obtained taking the best condition dataset into account. The estimated yield was validated with ground sample data; the results are shown in Figure 6.

Table 3 reports yield values recorded and estimated during the 2 years trial. In the 2017 season, the average production per plant in different vigor zones was calculated both with traditional ground measurements (HV = 803.7 ± 356.9, LV = 371.9 ± 202.0) and total cluster weight per vine detected by UAV (HV = 682.7 ± 279.6, LV = 323.0 ± 165.1). The yield estimation in the 2018 season confirmed the results of the previous year with a higher value both in ground measurements (HV = 2838.1 ± 1346.4, LV = 1559.2 ± 1066.4) and UAV estimation (HV = 2602.8 ± 1339.4, LV = 1315.7 ± 605.0). The results related to yield data estimation in worst condition evaluated during 2017 season confirm the need of line of sight to apply this methodology.

**Table 3:** Yield values measured by ground sampling and estimated from UAV in 2017 (W and B conditions) and 2018 (B condition).

	HV_2017 W	LV_2017 W	HV_2017 B	LV_2017 B	HV_2018 B	LV_2018 B
Measured yield (g/vine)	803.7 ± 356.9	371.9 ± 202.0	803.7 ± 356.9	371.9 ± 202.0	2838.1 ± 1346.4	1559.2 ± 1066.4
Estimated yield (g/vine)	324.4 ± 189.8	80.8 ± 102.5	682.7 ± 279.6	323.0 ± 165.1	2602.8 ± 1339.4	1315.7 ± 605.0
Accuracy (%)	40.1	22.2	84.9	86.9	91.7	84.4


## Discussion

Remote sensing images acquired during June by a UAV platform equipped with a multispectral camera allowed the assessment of spatial variation in terms of vigor in the experimental vineyard in both seasons. As reported by Romboli et al. (2017), the end of June is a good time to characterize vineyard vigor variability, and the map produced by the flight campaign in August confirmed the variability within the vineyard detected during the preliminary flight in June (Figure 4).

The results of the experimentation showed that the proposed methodology has difficulty discriminating green clusters within the canopy. As reported in Grocholsky et al. (2011), the clusters detected with minimal number of false negatives were due to unripe grapes and small clusters related to lateral shoot production. However, the exclusion of unripe clusters during the evaluation of vineyard yield potential gives added value to the effectiveness of the methodology. Unripe clusters cause a product quality loss by conferring a series of sensory characteristics and often producing astringent, bitter and low-alcohol wines (Peyrot des Gachons and Kennedy, 2003; Canals et al., 2005;Kontoudakis et al., 2011).

The cluster detection approach showed a poor performance in the worst condition dataset, substantially due to the leaf cover that blocked line of sight of the cluster, so the yield analysis was only performed on the best dataset. However, fruit zone defoliation is a widely used canopy management practice to improve light exposure for grape quality (Reynolds et al., 1986; Bergqvist et al., 2001; Downey et al., 2006; Cohen et al., 2012; Romboli et al., 2017). Defoliation at the beginning of veraison could cause higher incidence of sunburn damage, but 2–3 weeks before harvest is a diffuse practice also in warm winemaking areas, providing a risk reduction of fungal attack on grape clusters due to improved air circulation, decreased humidity and better penetration of fungicide sprays (Pieri and Fermaud, 2005; Sabbatini and Howell, 2010, Noyce et al., 2016). In that sense, our study takes into account a partial defoliation treatment only on the morning side (north-east) of the canopy, aiming to prevent sunburn and obtain the positive effects of this canopy management.

For the UAV yield estimation results, we combined clusters detected with cluster weight calculated on the basis of cluster dimensions extracted by pixel counts from high resolution images and cluster weight observed by ground measurements in a commercial vineyard in Italy. At the end of 2017 season, the UAV yield prediction approach was evaluated through the comparison of yield ground measurements on a large sample of vines in each vigor zone and data extrapolated from UAV images on a restricted number of vines in each zone. Correlation between the cluster segmentation approach and ground truth measurements, on 2017 data, was used to estimate yield from 2018 images analysis and then compared with traditional field measurements. The yield calculated from UAV images provided high accuracy (over 84.4% in both years) following the strong variability within the vineyard and identifying almost double yield in HV than LV zone, as clearly shown in biomass sampling data for both years. The yield data showed a great inter-annual variability due to the 2 years being completely different. The 2017 season was extremely hot and dry compared to 2018, and grape production in terms of cluster number and weight, resulted exceptionally lower. A direct consequence was less leaf coverage and a greater cluster separation in the 2017 season, which favored the method performance compared to the survey in 2018.

An accurate estimation of the yield several weeks before harvesting by a fast and non-destructive method, such as the one described in this paper, can provide very valuable information for the farmer for canopy management decisions, such as grape trimming, as well as for harvest planning (Diago et al., 2012). The proposed methodology is a good alternative to traditional measurement, due to its accuracy and relative speed and would provide the farmer with a promising tool for yield prediction in a fast and precise way. In recent years, innovative solutions have been proposed for grape image analysis based on lab (Diago et al., 2015; Tello et al., 2016) or on-the-go measurements (Aquino et al., 2018). Those methods are precise as the result of a proximal sensing approach, but are weak in terms of timing, which plays a key role in agriculture management. Diago et al. (2015) found that the best results (*R*^2^ between 69 and 95% in berry detection and between 65 and 97% in cluster weight estimation) were achieved using four images and the Canny algorithm. The model’s capacity based on image analysis to predict berry weight was 84%. Tello et al. (2016) studied cluster length, width and elongation by 2D image analysis and found significant and strong correlations with the manual methods with *r* = 0.959, 0.861, and 0.852, respectively. Aquino et al. (2018) applied mathematical morphology and a pixel classification method, which yielded overall average Recall and Precision values of 0.876 and 0.958, respectively. The lab approach is very time-consuming due to the need for destructive grape sampling in the field and transport to the lab, followed by image acquisition and analysis. Regarding on-the-go monitoring methods, the camera moving along the inter-row must be very close to the side of the canopy, providing an image with high resolution but related to very low canopy area. Consequently, in order to monitor a reasonable number of vines it is necessary acquire a large number of images, which causes a massive time increase for data elaboration. The advantage of the UAV approach is that working at a greater distance from the target vines it can acquire up to 10 plants in one image, at the same time providing enough resolution to correctly discriminate the clusters within the canopy. Moreover, the ground solution is dependent on terrain conditions; in fact, wet, sloping, ploughed or uneven soil could affect linear advancement of the platform and therefore image quality. Our methodology works well with vertical shoot position, which is a common and widely used trellis system due to its many good points: compatibility with vineyard mechanization, suitability for many grape varieties, fungal disease risk reduction by allowing good air circulation and light exposure.

The best results obtained depended on flight planning for correct image acquisition (flight altitude, gimbal angle, speed, image frequency acquisition, etc.) and sunlight condition (solar angle, light and shadows). This could be expected given differences in grape hue, color, size and cluster compactness, and because these differences were accentuated by the level of ripeness at the time of image acquisition. All these features greatly influenced the algorithms for contour detection.

A cost-benefit analysis was conducted for three ideal vineyard sizes and two scenarios: (i) accounting for a contractor service, thus not including the cost of purchasing a UAV and related maintenance costs or hiring an agronomist, (ii) assuming the cost of UAV+camera (Table 4).

**Table 4:** Category costs for traditional ground and UAV in field yield monitoring.

	Area (ha)	Survey time (h)	Survey cost ($)	Elaboration time (h)	Elaboration cost ($)	Time (h)	UAV ($)	Total Cost ($)	Cost excluding UAV ($)
Ground	5	2.1	33.6	0.2	2.7	2.3		36.3	36.3
	10	4.2	67.2	0.3	5.3	4.6		72.4	72.4
	50	20.8	332.8	1.7	26.7	22.4		359.6	359.6
UAV	5	0.2	4.8	0.8	16.0	1.0	206.7	227.5	20.8
	10	0.4	9.6	1.7	34.0	2.1	206.7	250.3	43.6
	50	1.7	40.8	8.3	166.0	10.0	206.7	413.5	206.8


Overall, on all three solutions (5, 10, and 50 ha) the use of UAV for implementing the proposed methodology appears to be the most time saving but is detrimental in terms of total cost due to the UAV and RGB camera purchase. This methodology requires a minimum farm size of 60 ha (data not shown) in order to depreciate the fixed-cost investments. However, where the field size is smaller than 60 ha, specialist UAV service providers, sharing of farming equipment and cooperative approaches may be suitable for use of the platform by different farmers (Zarco-Tejada et al., 2014). In fact, excluding platform and camera purchase, the final cost is in favor of using UAV. For the three spatial scales analyzed, savings are always slightly less than 50% compared to the cost of the traditional methodology. Although the error of the proposed methodology cannot be assessed in the cost analysis, nor the error relating to manual pre-harvest inspection, it should be noted that this methodology is able to capture the variability, in terms of production, within the vineyard and between the 2 years of analysis, with a certain tendency to underestimate the yield. It should also be considered that traditional survey methods are labor intensive and subject to observer bias (Zhou et al., 2018), while the automated methodology could reduce the bias and help breeders in crop yield phenotyping and farmers to be more efficient in crop planning, reducing labor costs and optimizing the available resources.

For our case study, a first flight was needed to identify zones with different vigor and recommend partial defoliation. It must be taken into account that the latter is a common technique that it is usually performed by the farmer some weeks before harvest, so there would be no additional cost. For the identification of the zones with different vigor the cost of a further flight could be avoided based on the direct experience of the farmer or consulting the freely available NDVI maps that are provided by satellite platforms such as Sentinel 2 or Landsat 8.

## Conclusion

The application of innovative technologies in field phenotyping such as UAV, digital image analysis tools and image interpretation techniques promises a methodology for yield and quality traits estimation in a vineyard in order to rapidly monitor representative zones in a large acreage, improve the quality of recording and minimize error variation between samples.

The methodology for cluster detection and image analysis described in this paper has proven to be a useful and reliable tool for yield assessment in a vineyard. The approach for image-acquisition and data elaboration is simple and low-cost as it only needs a commercial RGB camera, a base level UAV platform and free image analysis software.

This study analyses the potential of UAV technology to estimate yield in a vineyard with high resolution RGB images in partially leaf removal and full ripe conditions several weeks before harvest. First, an unsupervised cluster detection approach was tested on two different datasets in worst (leaves cover, shaded fruit) and best condition (partially defoliated and directly illuminated fruit) in both high and low vigor zones during the 2017 season. A linear correlation between yield per vine and ground truth measurements in different vigor zones was then performed. The correlation parameters were applied to the 2018 dataset, providing interesting yield prediction performance with about 12% under-estimation. Further tests are necessary to extend and confirm the preliminary results obtained from this study, in terms of camera setting (exposure, etc.), optimal environmental conditions (time of day, angle of incidence of the sun, etc.), setting of the gimbal (camera angle of inclination), flight parameters (speed, flight quote, overlap, etc.) and crop features (more varieties and different crops, training system, phenological stage, crop management, plant spacing, etc.). However, the data are decidedly encouraging. Indeed, given the continuous technological development in image analysis tools, cameras and UAV performances, it will be possible to improve the methodology efficacy in terms of accuracy, times for data acquisition and analysis, and costs. This optimized tool could be a useful support for both phenotyping research and agronomic management.

## Author Contributions

SDG and AM designed the experiment and coordinated the activity. SDG, AM, PC, and AB performed the UAV data acquisition. SDG, AM, PC, and PT data analysis and wrote the manuscript. All authors read and approved the final manuscript.

## Conflict of Interest Statement

The authors declare that the research was conducted in the absence of any commercial or financial relationships that could be construed as a potential conflict of interest.

## Footnotes

1 www.tetracam.com

2 www.agisoft.ru

3 www.qgis.org

4 www.mathworks.com

5 www.payscale.com (accessed September 09, 2018)

## References

[B1] Aquino A. Diago M. P. Millan B. Tardaguila J. (2017). A new methodology for estimating the grapevine-berry number per cluster using image analysis. *Biosyst. Eng.* 156 80–95. 10.1016/j.biosystemseng.2016.12.011

[B2] Aquino A. Millan B. Diago M. P. Tardaguila J. (2018). Automated early yield prediction in vineyards from on-the-go image acquisition. *Comput. Electron. Agric.* 144 26–36. 10.1016/j.compag.2017.11.026

[B3] Aquino A. Millan B. Gutierrez S. Tardaguila J. (2015). Grapevine flower estimation by applying artificial vision techniques on images with uncontrolled scene and multi-model analysis. *Comput. Electron. Agric.* 119 92–104. 10.1016/j.compag.2015.10.009

[B4] Bellvert J. Marsal J. Girona J. Gonzalez-Dugo V. Fereres E. Ustin S. L. (2016). Airborne thermal imagery to detect the seasonal evolution of crop water status in peach, nectarine and saturn peach orchards. *Remote Sens.* 8 1–17. 10.3390/rs8010039

[B5] Bergqvist J. Dokoozlian N. Ebisuda N. (2001). Sunlight exposure and temperature effects on berry growth and composition of cabernet sauvignon and grenache in the central San Joaquin Valley of California. *Am. J. Enol. Vitic.* 52 1–7.

[B6] Bindon K. Kassara S. Cynkar W. Robinson E. Scrimgeor N. Smith P. A. (2014). Comparison of extraction protocols to determine differences in wine-extractable tannin and anthocyaninin *Vitis vinifera* L. cv. shiraz and cabernet sauvignon grapes. *J. Agric. Food Chem.* 62 4558–4570. 10.1021/jf5002777 24773241

[B7] Cabezas J. A. Cervera M. T. Ruiz-Garcia L. Carreno J. Martinez-Zapater J. M. (2006). A genetic analysis of seed and berry weight in grapevine. *Genome* 49 1572–1585. 10.1139/g06-122 17426772

[B8] Canals R. Llaudy M. C. Valls J. Canals J. M. Zamora F. (2005). Influence of ethanol concentration on the extraction of colour and phenolic compounds from the skin and seeds of Tempranillo grapes at different stages of ripening. *J. Agric. Food Chem.* 53 4019–4025. 10.1021/jf047872v 15884833

[B9] Cohen Y. Alchanatis V. Prigojin A. Levi A. Soroker V. (2012). Use of aerial thermal imaging to estimate water status of palm trees. *Precis. Agric.* 3 123–140. 10.1007/s11119-011-9232-7

[B10] Costa C. Antonucci F. Pallottino F. Aguzzi J. Sun D. Menesatti P. (2011). Shape analysis of agricultural products: a review of recent research advances and potential application to computer vision. *Food Bioprocess Technol.* 4 673–692. 10.1007/s11947-011-0556-0

[B11] Costa-Ferreira A. M. Germain C. Homayouni S. Da Costa J. P. Grenier G. Marguerit E. (2007). “Transformation of high resolution aerial images in vine vigour maps at intra-block scale by semiautomatic image processing,” in *Proceedings of the XV International Symposium GESCO*, Porec, 1372–1381.

[B12] Costantini L. Battilana J. Lamaj F. Fanizza G. Grando M. (2008). Berry and phenology-related traits in grapevine (*Vitis vinifera* L.): from quantitative trait loci to underlying genes. *BMC Plant Biol.* 8:38. 10.1186/1471-2229-8-38 18419811 PMC2395262

[B13] Cubero S. Aleixos N. Moltó E. Gómez-Sanchis J. Blasco J. (2011). Advances in machine vision applications for automatic inspection and quality evaluation of fruits and vegetables. *Food Bioprocess Technol.* 4 487–504. 10.1007/s11947-010-0411-8

[B14] Cubero S. Diago M. P. Blasco J. Tardaguila J. Millan B. Aleixos N. (2014). A new method for pedicel/peduncle detection and size assessment of grapevine berries and other fruits by image analysis. *Biosyst. Eng.* 117 62–72. 10.1016/j.biosystemseng.2013.06.007

[B15] Di Gennaro S. F. Battiston E. Di Marco S. Facini O. Matese A. Nocentini M. (2016). Unmanned aerial vehicle (UAV)-based remote sensing to monitor grapevine leaf stripe disease within esca complex in vineyard. *Phytopathol. Mediterr.* 55 262–275 10.14601/Phytopathol_Mediterr-18312

[B16] Diago M. P. Correa C. Millán B. Barreiro P. Valero C. Tardaguila J. (2012). Grapevine yield and leaf area estimation using supervised classification methodology on rgb images taken under field conditions. *Sensors* 12 16988–17006. 10.3390/s121216988 23235443 PMC3571822

[B17] Diago M. P. Sanz-Garcia A. Millan B. Blasco J. Tardaguila J. (2014). Assessment of flower number per inflorescence in grapevine by image analysis under field conditions. *J. Sci. Food Agric.* 94 1981–1987. 10.1002/jsfa.6512 24302287

[B18] Diago M. P. Tardaguila J. Aleixos N. Millan B. Prats-Montalban J. M. Cubero S. (2015). Assessment of cluster yield components by image analysis. *J. Sci. Food Agric.* 95 1274–1282. 10.1002/jsfa.6819 25041796

[B19] Downey M. O. Dokoozlian N. K. Krstic M. P. (2006). Cultural practice and environmental impacts on the flavonoid composition of grapes and wine: a review of recent research. *Am. J. Enol. Viticult.* 57 257–268.

[B20] Dunn G. M. Martin S. R. (2004). Yield prediction from digital image analysis: a technique with potential for vineyard assessments prior to harvest. *Aust. J. Grape Wine R.* 10 196–198. 10.1111/j.1755-0238.2004.tb00022.x

[B21] Eurostat (2018). *The European Farm structure survey, Eurostat Database.*

[B22] Fanizza G. Lamaj F. Costantini L. Chabane R. Grando M. S. (2005). QTL analysis for fruit yield components in table grapes (*Vitis vinifera*). *Theor. Appl. Genet.* 111 658–664. 10.1007/s00122-005-2016-6 15995866

[B23] Fiorillo E. Crisci A. De Filippis T. Di Gennaro S. F. Di Blasi S. Matese A. (2012). Airborne high-resolution images for grape classification: changes in correlation between technological and late maturity in a Sangiovese vineyard in Central Italy. *Aust. J. Grape Wine R.* 18 80–90. 10.1111/j.1755-0238.2011.00174.x

[B24] Gago J. Douthe C. Coopman R. E. Gallego P. P. Ribas-Carbo M. Flexas J. (2015). UAVs challenge to assess water stress for sustainable agriculture. *Agric. Water Manage.* 153 9–19. 10.1016/j.agwat.2015.01.020

[B25] Gebhardt S. Schellberg J. Lock R. Kühbauch W. (2006). Identification of broad-leaved dock (*Rumex obtusifolius* L.) on grassland by means of digital image processing. *Precis. Agric.* 7 165–178. 10.1007/s11119-006-9006-9

[B26] Grocholsky B. Nuske S. Aasted M. Achar S. Bates T. (2011). “A camera and laser system for automatic vine balance assessment,” in *Proceedings of the ASABE Annual International Meeting*, Louisville, KY.

[B27] Hao M. Yu H. Li D. (2016). The measurement of fish size by machine vision-a review. *IFIP Adv. Inform. Commun. Technol.* 479 15–32. 10.1007/978-3-319-48354-2_2

[B28] Herzog K. Roscher R. Wieland M. Kicherer A. Läbe T. Förstner W. (2014). Initial steps for high-throughput phenotyping in vineyards. *Vitis* 53 1–8.

[B29] Igathinathane C. Pordesimo L. O. Columbus E. P. Batchelor W. D. Methuku S. R. (2008). Shape identification and particles size distribution from basic shape parameters using ImageJ. *Comput. Electron. Agric.* 63 168–182. 10.1016/j.compag.2008.02.007

[B30] International Organisation of Vine and Wine [OIV] (2007). *World Vitivinicultural Statistics 2007 – Structure of the World Vitivinicultural Industry 2007.* Available at: http://news.reseauconcept.net/images/oiv_uk/Client/Statistiques_commentaires_annexes_2007_EN.pdf (accessed May 15, 2018).

[B31] International Organisation of Vine and Wine [OIV] (2017). *World Vitivinicultural Situation - OIV Statistical report on World Vitivinicultural.* Available at: http://www.oiv.int/js/lib/pdfjs/web/viewer.html?file=/public/medias/5479/oiv-en-bilan-2017.pdf (accessed May 15, 2018).

[B32] Ivorra E. Sánchez A. J. Camarasa J. G. Diago M. P. Tardáguila J. (2015). Assessment of grape cluster yield components based on 3D descriptors using stereo vision. *Food Control* 50 273–282. 10.1016/j.foodcont.2014.09.004

[B33] Kicherer A. Roscher R. Herzog K. Förstner W. Töpfer R. (2015). Image based evaluation for the detection of cluster parameters in grapevine. *Acta Hortic.* 1082 335–340. 10.17660/actahortic.2015.1082.46

[B34] Kicherer A. Roscher R. Herzog K. Šimon S. Förstner W. Töpfer R. (2013). BAT (berry analysis tool): a high-throughput image interpretation tool to acquire the number, diameter, and volume of grapevine berries. *Vitis* 52 129–135.

[B35] Kontoudakis N. Esteruelas M. Fort F. Canals J. M. Freitas V. Zamora F. (2011). Influence of the heterogeneity of grape phenolic maturity on wine composition and quality. *Food Chem.* 124 767–774. 10.1016/j.foodchem.2010.06.093

[B36] Ling P. P. Ruzhitsky V. N. (1996). Machine vision techniques for measuring the canopy of tomato seedling. *J. Agric. Eng. Res.* 65 85–95. 10.1006/jaer.1996.0082

[B37] Liu Y. Mu X. Wang H. Yan G. (2012). A novel method for extracting green fractional vegetation cover from digital images. *J. Veg. Sci.* 23 406–418. 10.1111/j.1654-1103.2011.01373.x

[B38] Matese A. Di Gennaro S. F. (2018). Practical applications of a multisensor UAV platform based on multispectral, thermal and rgb high resolution images in precision viticulture. *Agriculture* 8:116 10.3390/agriculture8070116

[B39] Matese A. Di Gennaro S. F. Berton A. (2016). Assessment of a canopy height model (CHM) in a vineyard using UAV-based multispectral imaging. *Int. J. Remote Sens.* 38 2150–2160. 10.1080/01431161.2016.1226002

[B40] Matese A. Toscano P. Di Gennaro S. F. Genesio L. Vaccari F. P. Primicerio J. (2015). Intercomparison of UAV, aircraft and satellite remote sensing platforms for precision viticulture. *Remote Sens.* 7 2971–2990. 10.3390/rs70302971

[B41] Miao Y. Xu M. Zhai P. (2012). GVF snake model based on the constraint of prior shape for overlapping grape image segmentation algorithm. *J. Inform. Comput. Sci.* 9 5865–5872.

[B42] Moallem P. Serajoddin A. Pourghassem H. (2017). Computer vision-based apple grading for golden delicious apples based on surface features. *Inform. Process. Agric.* 4 33–40. 10.1016/j.inpa.2016.10.003

[B43] Noyce P. Steel C. Harper J. Wood M. R. (2016). The basis of defoliation effects on reproductive parameters in vitis vinifera l. cv. chardonnay lies in the latent bud. *Am. J. Enol. Viticult.* 67 199–205. 10.5344/ajev.2015.14051

[B44] Nuske S. Achar S. Bates T. Narasimhan S. Singh S. (2011). “Yield estimation in vineyards by visual grape detection,” in *Proceedings of the IEEE/RSJ International Conference on Intelligent Robots and Systems*, San Francisco, CA, 2352–2358.

[B45] Nuske S. Wilshusen K. Achar S. Yoder L. Narasimhan S. Singh S. (2014). Automated visual yield estimation in vineyards. *J. Field Robot.* 31 837–860. 10.1002/rob.21541

[B46] Peyrot des Gachons C. Kennedy J. A. (2003). Direct method for determining seed and skin proanthocyanidin extraction in red wine. *J. Agric. Food Chem.* 51 5877–5881. 10.1021/jf034178r 13129288

[B47] Pieri P. Fermaud M. (2005). Effects of defoliation on temperature and wetness of grapevine berries. *Acta Hortic.* 689 109–116 10.17660/ActaHortic.2005.689.9

[B48] Poblete-Echeverría C. Olmedo G. F. Ingram B. Bardeen M. (2017). Detection and segmentation of vine canopy in ultra-high spatial resolution rgb imagery obtained from unmanned aerial vehicle (UAV): a case study in a commercial vineyard. *Remote Sens.* 9:268 10.3390/rs9030268

[B49] Pôças I. Paço T. A. Paredes P. Cunha M. Pereira L. S. (2015). Estimation of actual crop coefficients using remotely sensed vegetation indices and soil water balance modelled data. *Remote Sens.* 7 2373–2400. 10.3390/rs70302373

[B50] Portales C. Ribes-Gomez E. (2015). An image-based system to preliminary assess the quality of grape harvest batches on arrival at the winery. *Comput. Ind.* 68 105–115. 10.1016/j.compind.2014.12.010

[B51] Pothen Z. Nuske S. T. (2016). Automated assessment and mapping of grape quality through image based color analysis. *IFAC-PapersOnLine* 49 72–78. 10.1016/j.ifacol.2016.10.0111

[B52] Reynolds A. G. Pool R. M. Mattick L. R. (1986). Influence of cluster exposure on fruit composition and wine quality of seyval blanc grapes. *Vitis* 25 85–95.

[B53] Romboli Y. Di Gennaro S. F. Mangani S. Buscioni G. Matese A. Genesio L. (2017). Vine vigour modulates bunch microclimate and affects the composition of grape and wine flavonoids: an unmanned aerial vehicle approach in a Sangiovese vineyard in Tuscany. *Aust. J. Grape Wine R.* 23 368–377. 10.1111/ajgw.12293

[B54] Roscher R. Herzog K. Kunkel A. Kicherer A. Töpfer R. Förstner W. (2014). Automated image analysis framework for high-throughput determination of grapevine berry sizes using conditional random fields. *Comput. Electron. Agric.* 100 148–158. 10.1016/j.compag.2013.11.008

[B55] Rouse J. W. Haas R. H. Schell J. A. Deering D. W. (1973). “Monitoring vegetation systems in the great plains with ERTS,” in *Proceedings of the 3rd ERTS Symposium*, Washington, DC, 309–317.

[B56] Sabbatini P. Howell G. S. (2010). Effects of early defoliation on yield, fruit composition, and harvest season cluster rot complex of grapevines. *HortScience* 45 1804–1808. 10.21273/HORTSCI.45.12.1804

[B57] Santesteban L. G. Di Gennaro S. F. Herrero-Langreo A. Miranda C. Royo J. B. Matese A. (2017). High-resolution UAV-based thermal imaging to estimate the instantaneous and seasonal variability of plant water status within a vineyard. *Agric. Water Manage.* 183 49–59. 10.1016/j.agwat.2016.08.026

[B58] Schneider C. A. Rasband W. S. Eliceiri K. W. (2012). NIH image to ImageJ: 25 years of image analysis. *Nat. Methods* 9 671–675. 10.1038/nmeth.2089 22930834 PMC5554542

[B59] Shrestha D. S. Steward B. L. Birrell S. J. (2004). Video processing for early stage maize plant detection. *Biosyst. Eng.* 89 119–129. 10.1016/j.biosystemeng.2004.06.007

[B60] Soltani M. Omid M. Alimardani R. (2015). Egg volume prediction using machine vision technique based on Pappus theorem and artificial neural network. *J. Food Sci. Technol.* 52 3065–3071. 10.1007/s13197-014-1350-6 25892810 PMC4397291

[B61] Song W. Mu X. Yan G. Huang S. (2015). Extracting the green fractional vegetation cover from digital images using a shadow-resistant algorithm (SHAR-LABFVC). *Remote Sens.* 7 10425–10443. 10.3390/rs70810425

[B62] Su Q. Kondo N. Li M. Sun H. Al Riza D. F. (2017). Potato feature prediction based on machine vision and 3D model rebuilding. *Comput. Electron. Agric.* 137 41–51. 10.1016/j.compag.2017.03.020

[B63] Sun W. Du Q. (2018). Graph-regularized fast and robust principal component analysis for hyperspectral band selection. *IEEE Trans. Geosci. Remote Sens.* 56 3185–3195. 10.1109/TGRS.2018.2794443

[B64] Tardaguila J. Blanco J. A. Poni S. Diago M. P. (2012). Mechanical yield regulation in winegrapes: comparison of early defoliation and crop thinning. *Aust. J. Grape Wine R.* 18 344–352. 10.1111/j.1755-0238.2012.00197.x

[B65] Tello J. Cubero S. Blasco J. Tardaguila J. Aleixos N. Ibáñez J. (2016). Application of 2D and 3D image technologies to characterise morphological attributes of grapevine clusters. *J. Sci. Food Agric.* 96 4575–4583. 10.1002/jsfa.7675 26910811

[B66] Tello J. Ibanez J. (2014). Evaluation of indexes for the quantitative and objective estimation of grapevine bunch compactness. *Vitis* 53 9–16.

[B67] Wycislo A. P. Clark J. R. Karcher D. E. (2008). Fruit shape analysis of Vitis using digital photography. *HortScience* 43 677–680. 10.21273/hortsci.43.3.677

[B68] Yang W. Wang S. Zhao X. Zhang J. Feng J. (2015). Greenness identification based on HSV decision tree. *Inform. Process. Agric.* 2 149–160. 10.1016/j.inpa.2015.07.003

[B69] Zarco-Tejada P. J. Hubbard N. Loudjani P. (2014). *Precision Agriculture: An Opportunity for EU Farmers -Potential Support With the CAP 2014-2020.* Brussels: European Parliament Directorate-General for Internal Policies, Policy Department B: Structural and Cohesion Policies.

[B70] Zhang D. Lillywhite K. D. Lee D. J. Tippetts B. J. (2014). Automatic shrimp shape grading using evolution constructed features. *Comput. Electron. Agric.* 100 116–122. 10.1016/j.compag.2013.11.009

[B71] Zhao T. Wang Z. Yang Q. Chen Y. (2017). “Melon yield prediction using small unmanned aerial vehicles,” in *Proceedings of the Autonomous Air and Ground Sensing Systems for Agricultural Optimization and Phenotyping II* (Anaheim, CA: SPIE), 1021808 10.1117/12.2262412

[B72] Zhou C. Liang D. Yang X. Yang H. Yue J. Yang G. (2018). Wheat ears counting in field conditions based on multi-feature optimization and TWSVM. *Front. Plant Sci.* 9:1024. 10.3389/fpls.2018.01024 30057587 PMC6053621

